# (*S*,*S*)-*N*,*N*′-Bis(1-carb­oxy-2-methyl­prop­yl)ethyl­enediammonium dihalide cyclo­penta­nol tetra­solvate (halide = bromide/chloride ≃ 1:12)

**DOI:** 10.1107/S1600536809006679

**Published:** 2009-02-28

**Authors:** Bojana B. Zmejkovski, Goran N. Kaluderović, Santiago Gómez-Ruiz, Tibor J. Sabo

**Affiliations:** a Department of Chemistry, Institute of Chemistry, Technology and Metallurgy, University of Belgrade, Studentski trg 14, 11000 Belgrade, Serbia; b Departamento de Química Inorgánica y Analítica, ESCET, Universidad, Rey Juan Carlos, 28933 Móstoles, Madrid, Spain; c Faculty of Chemistry, University of Belgrade, Studentski trg 12-14, PO Box 158, 11000 Belgrade, Serbia

## Abstract

In the crystal structure of the title compound, C_12_H_26_N_2_O_4_^2+^·2(Br_0.085_Cl_0.915_)^−^·4C_5_H_9_OH, the complete cation is generated by crystallographic twofold symmetry. Contamination of the chloride counter-anion with bromide occured during the preparation, due to the use of 1,2-dibromo­ethane. One of the solvent mol­ecules is disordered, with occupancies 0.53 (3):0.47 (3). The crystal packing is stabilized by an infinite two dimensional ⋯*X*⋯H—N—H⋯*X*⋯ hydrogen-bonding network (*X*: Br^−^/Cl^−^ ≃ 1:12). In addition, O—H⋯*X* and O—H⋯O hydrogen bonds involving solvent mol­ecules are observed.

## Related literature

For dihydro­chloride salts of the analog ethyl­enediamine-*N*,*N*′-diacetic acid and ethyl­enediamine-*N*,*N*′-di-3-propionic acid, see: Mistryukov *et al.* (1987 ▶); Shkol’nikova *et al.* (1989 ▶, 1990 ▶, 1992 ▶). For bond lengths and angles in ethyl­enediammonium-*N*,*N*′-di-3-propanoic acid dichloride and similar compounds, see: Kaluderović *et al.* (2004 ▶, 2007 ▶). For the synthesis, see: Schoenberg *et al.* (1968 ▶).
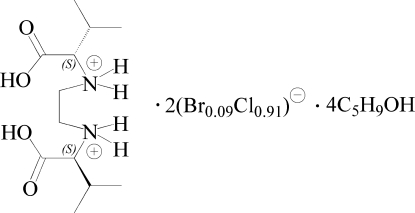


## Experimental

### 

#### Crystal data

C_12_H_26_N_2_O_4_^2+^·2(Br_0.09_Cl_0.91_)^−^·4C_5_H_10_O
*M* *_r_* = 685.41
Monoclinic, 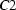

*a* = 21.2037 (5) Å
*b* = 5.2166 (1) Å
*c* = 17.2517 (5) Å
β = 97.037 (2)°
*V* = 1893.86 (8) Å^3^
*Z* = 2
Mo *K*α radiation
μ = 0.39 mm^−1^
*T* = 130 K
0.7 × 0.04 × 0.04 mm

#### Data collection

Oxford Diffraction CCD Oxford Xcalibur S diffractometer
Absorption correction: multi-scan (*CrysAlis RED*; Oxford Diffraction, 2009 ▶) *T*_min_ = 0.981, *T*_max_ = 0.985
28298 measured reflections
5795 independent reflections
4851 reflections with *I* > 2σ(*I*)
*R*_int_ = 0.035

#### Refinement

*R*[*F* ^2^ > 2σ(*F* ^2^)] = 0.042
*wR*(*F* ^2^) = 0.104
*S* = 0.98
5795 reflections
232 parameters
92 restraints
H atoms treated by a mixture of independent and constrained refinement
Δρ_max_ = 0.62 e Å^−3^
Δρ_min_ = −0.37 e Å^−3^
Absolute structure: Flack (1983 ▶), 2602 Friedel pairs
Flack parameter: −0.04 (2)

### 

Data collection: *CrysAlisPro* (Oxford Diffraction, 2009 ▶); cell refinement: *CrysAlisPro*; data reduction: *CrysAlisPro*; program(s) used to solve structure: *SHELXS97* (Sheldrick, 2008 ▶); program(s) used to refine structure: *SHELXL97* (Sheldrick, 2008 ▶); molecular graphics: *ORTEP-3* (Farrugia, 1997 ▶); software used to prepare material for publication: *SHELXL97*.

## Supplementary Material

Crystal structure: contains datablocks I, global. DOI: 10.1107/S1600536809006679/fj2196sup1.cif

Structure factors: contains datablocks I. DOI: 10.1107/S1600536809006679/fj2196Isup2.hkl

Additional supplementary materials:  crystallographic information; 3D view; checkCIF report

## Figures and Tables

**Table 1:** Hydrogen-bond geometry (Å, °)

*D*—H⋯*A*	*D*—H	H⋯*A*	*D*⋯*A*	*D*—H⋯*A*
N1—H1*N*⋯*X*1	0.88 (2)	2.37 (2)	3.253 (2)	175 (2)
N1—H2*N*⋯*X*1^i^	0.93 (2)	2.32 (2)	3.209 (2)	161 (2)
O1—H1*O*⋯O4^ii^	0.95 (4)	2.50 (4)	3.446 (3)	172 (5)
O4—H4*O*⋯O3	0.86 (3)	1.89 (3)	2.728 (2)	165 (3)
O3—H3*O*⋯Cl1	0.94 (3)	2.29 (3)	3.204 (2)	163 (2)
O3—H3*O*⋯Br1	0.94 (3)	2.29 (3)	3.204 (2)	163 (2)

Symmetry codes: (i) 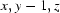; (ii) 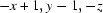. *X*1 is the disordered Cl/Br atom.

**Fig. 1.**
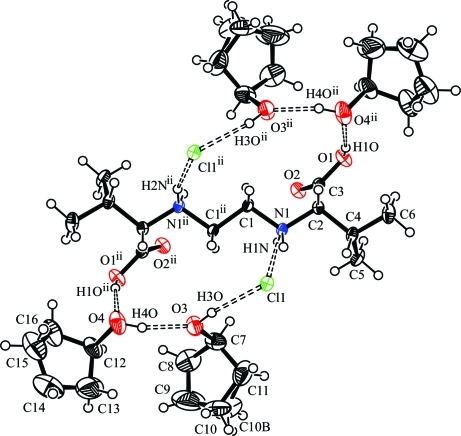
ORTEP representation of [(H4eddv)X2].4C5H9OH. The structure contains a 1:12 Br/Cl (X) disorder. The figure displays the Cl-part of this disorder (Cl1). Displacement ellipsoids are plotted at the 50% probability level and H atoms are shown as small spheres of arbitrary radii.

**Fig. 2.**
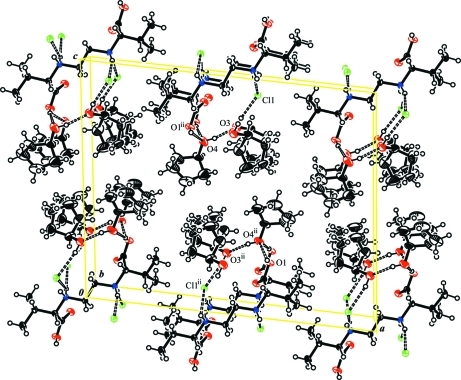
Network of H-bonding.

## supplementary crystallographic information

### Comment

Dihydrochloride salts of the analog ethylenediamine-*N,N'*-diacetic acid and ethylenediamine-*N,N'*-di-3-propionic acid are reported in the literature, see (Shkol'nikova *et al.*, 1989; Shkol'nikova *et al.*, 1990; Shkol'nikova *et al.*, 1992; Mistryukov *et al.*, 1987).

Crude (*S,S*)-ethylenediammonium-*N,N'*-di-2-(3-methyl)-butanoic acid dihalide, [(H_4_eddv)X_2_], obtained from the reaction of L-valine and 1,2-dibromethane (Schoenberg *et al.*,1968), was used for the synthesis of dicyclopentyl ester. The title compound is isolated from the mother liquor as a mixture of Cl and Br salts. The structure consists of several species: one dicationic, C_12_H_26_N_2_O_4_^2+^, 0.17 Br^–^ and 1.83 Cl^–^ anions and four cyclopentanol molecules (Fig. 1). Bond lengths and angles are comparable with those of ethylenediammonium-*N*,*N'*-di-3-propanoic acid dichloride and similar compounds (Kaluđerović *et al.*, 2004, 2007). All of the mentioned species are stabilizing the structure by intramolecular and intermolecular H-bonds (Table 1). The solvent molecules are involved in hydrogen bonding, through O4–H4O···O3 atoms (Fig. 2). Furthermore, the H3O atom bonded to O3 is participating in hydrogen bonding with X atom (X: Br^–^/Cl^–^≈ 1:12), which is on the other side interacting *via* hydrogen bond with the H1N–N1 moiety. The cyclopentyl rings are in envelope conformations.

### Experimental

(*S,S*)-ethylenediammonium-*N,N'*-di-2-(3-methyl)-butanoic acid dihalide is obtained as earlier described in literature (Schoenberg *et al.*,1968), by combining the solutions of L-valine and 1,2-dibromoethane. The title compound is obtained unintentionally. The goal was to synthesize a dicyclopentyl ester of (*S,S*)-ethylenediammonium-*N,N'*-di-2-(3-methyl)-butanoic acid dichloride. Thionyl chloride (4.0 ml, 55 mmol) was introduced into a flask containing cyclopentanol (50 ml, anhydrous conditions) over 1 h. After that (*S,S*)-ethylenediammonium-*N,N'*-di-2-(3-methyl)-butanoic acid dihalide (calculated for X=Cl: 2.0 g, 6.00 mmol) was added to the flask and the suspension was refluxed 16 h. The mixture was filtered off and the filtrate was left for a few days at 4 °C yielding crystals suitable for X-ray measurements.

### Refinement

The H atoms connected to the nitrogen and oxygen atoms were found in difference maps and yielded reasonable bond lengths and angles (O—H bond length: 0.86 (3) – 0.95 (2) Å); N—H bond length: 0.88 (2) and 0.93 (2) Å), all other H atoms were positioned geometrically and treated as riding, with C—H bonding lengths constrained to 0.98–1.00 Å. The two positions of the disordered Cl- *versus* Br-atoms were determined from the difference map and refined anisotropically with occupancies of 0.915 (Cl) and 0.085 (Br).

### Crystal data

**Table:**

C_12_H_26_N_2_O_4_^2+^·2(Br_0.09_Cl_0.91_^−^)·4(C_5_H_10_O)	*F*(000) = 745.4
*M**_r_* = 685.41	*D*_x_ = 1.202 Mg m^−^^3^
Monoclinic, *C*2	Mo *K*α radiation, λ = 0.71073 Å
Hall symbol: C 2y	Cell parameters from 12428 reflections
*a* = 21.2037 (5) Å	θ = 2.9–32.3°
*b* = 5.2166 (1) Å	µ = 0.39 mm^−^^1^
*c* = 17.2517 (5) Å	*T* = 130 K
β = 97.037 (2)°	Needles, colourless
*V* = 1893.86 (8) Å^3^	0.7 × 0.04 × 0.04 mm
*Z* = 2	

### Data collection

**Table:**

Oxford Diffraction CCD Oxford Xcalibur S diffractometer	5795 independent reflections
graphite	4851 reflections with *I* > 2σ(*I*)
Detector resolution: 16.356 pixels mm^-1^	*R*_int_ = 0.035
ω and φ scans	θ_max_ = 30.5°, θ_min_ = 2.9°
Absorption correction: multi-scan (*CrysAlis RED*; Oxford Diffraction, 2009)	*h* = −30→30
*T*_min_ = 0.981, *T*_max_ = 0.985	*k* = −7→7
28298 measured reflections	*l* = −24→24

### Refinement

**Table:**

Refinement on *F*^2^	Secondary atom site location: difference Fourier map
Least-squares matrix: full	Hydrogen site location: inferred from neighbouring sites
*R*[*F*^2^ > 2σ(*F*^2^)] = 0.042	H atoms treated by a mixture of independent and constrained refinement
*wR*(*F*^2^) = 0.104	*w* = 1/[σ^2^(*F*_o_^2^) + (0.0675*P*)^2^] where *P* = (*F*_o_^2^ + 2*F*_c_^2^)/3
*S* = 0.98	(Δ/σ)_max_ = 0.001
5795 reflections	Δρ_max_ = 0.62 e Å^−^^3^
232 parameters	Δρ_min_ = −0.37 e Å^−^^3^
92 restraints	Absolute structure: Flack (1983), 2602 Friedel pairs
Primary atom site location: structure-invariant direct methods	Flack parameter: −0.04 (2)

### Special details

**Table:**

Geometry. All esds (except the esd in the dihedral angle between two l.s. planes) are estimated using the full covariance matrix. The cell esds are taken into account individually in the estimation of esds in distances, angles and torsion angles; correlations between esds in cell parameters are only used when they are defined by crystal symmetry. An approximate (isotropic) treatment of cell esds is used for estimating esds involving l.s. planes.
Refinement. Refinement of F^2^ against ALL reflections. The weighted R-factor wR and goodness of fit S are based on F^2^, conventional R-factors R are based on F, with F set to zero for negative F^2^. The threshold expression of F^2^ > 2sigma(F^2^) is used only for calculating R-factors(gt) etc. and is not relevant to the choice of reflections for refinement. R-factors based on F^2^ are statistically about twice as large as those based on F, and R- factors based on ALL data will be even larger.

### Fractional atomic coordinates and isotropic or equivalent isotropic displacement parameters (Å^2^)

**Table:**

	*x*	*y*	*z*	*U*_iso_*/*U*_eq_	Occ. (<1)
Cl1	0.597627 (15)	0.42698 (6)	−0.072483 (19)	0.02203 (10)	0.914 (2)
Br1	0.597627 (15)	0.42698 (6)	−0.072483 (19)	0.02203 (10)	0.086 (2)
O1	0.63329 (7)	0.1414 (3)	0.23276 (8)	0.0340 (3)	
O2	0.58689 (6)	0.3389 (2)	0.12648 (7)	0.0267 (3)	
O3	0.52079 (7)	0.6832 (3)	−0.22443 (9)	0.0422 (4)	
O4	0.40459 (9)	0.5428 (4)	−0.29775 (10)	0.0513 (5)	
N1	0.58713 (5)	−0.0783 (3)	0.03571 (7)	0.0172 (2)	
C1	0.51770 (7)	−0.1047 (4)	0.04083 (9)	0.0201 (3)	
H1A	0.5029	0.0396	0.0712	0.024*	
H1B	0.5094	−0.2666	0.0677	0.024*	
C2	0.62661 (6)	−0.0867 (4)	0.11390 (8)	0.0184 (3)	
H2	0.6129	−0.2374	0.1435	0.022*	
C3	0.61327 (8)	0.1557 (3)	0.15757 (10)	0.0205 (3)	
C4	0.69721 (7)	−0.1218 (3)	0.10265 (10)	0.0224 (4)	
H4	0.6993	−0.2621	0.0636	0.027*	
C5	0.72565 (9)	0.1159 (4)	0.07002 (13)	0.0325 (4)	
H5A	0.7252	0.2571	0.1074	0.049*	
H5B	0.7006	0.1637	0.0206	0.049*	
H5C	0.7696	0.0805	0.061	0.049*	
C6	0.73642 (9)	−0.2077 (4)	0.17799 (12)	0.0352 (5)	
H6A	0.7802	−0.2417	0.168	0.053*	
H6B	0.718	−0.3643	0.1971	0.053*	
H6C	0.7364	−0.0724	0.2174	0.053*	
C7	0.56429 (12)	0.8890 (5)	−0.23271 (13)	0.0457 (6)	
H7	0.5722	0.9917	−0.1835	0.055*	
C8	0.53654 (16)	1.0530 (6)	−0.29992 (19)	0.0644 (8)	
H8A	0.4897	1.0364	−0.3083	0.077*	
H8B	0.5477	1.2354	−0.2903	0.077*	
C9	0.5648 (3)	0.9555 (15)	−0.3675 (2)	0.137 (2)	
H9A	0.5313	0.8773	−0.405	0.164*	
H9B	0.5837	1.0998	−0.394	0.164*	
C10	0.6126 (5)	0.7700 (16)	−0.3447 (4)	0.065 (3)	0.53 (3)
H10A	0.6514	0.8074	−0.3693	0.078*	0.53 (3)
H10B	0.5974	0.5964	−0.3608	0.078*	0.53 (3)
C10B	0.6295 (7)	0.875 (6)	−0.3364 (7)	0.120 (6)	0.47 (3)
H10C	0.6441	0.7349	−0.3687	0.144*	0.47 (3)
H10D	0.6594	1.0202	−0.3365	0.144*	0.47 (3)
C11	0.62670 (13)	0.7854 (7)	−0.2568 (2)	0.0678 (8)	
H11A	0.6369	0.6143	−0.2337	0.081*	
H11B	0.6625	0.9036	−0.2408	0.081*	
C12	0.38374 (13)	0.6636 (5)	−0.37004 (13)	0.0477 (6)	
H12	0.3969	0.8479	−0.3686	0.057*	
C13	0.40779 (19)	0.5284 (12)	−0.4380 (2)	0.115 (2)	
H13A	0.446	0.4245	−0.4205	0.138*	
H13B	0.4182	0.6525	−0.478	0.138*	
C14	0.3485 (2)	0.3475 (7)	−0.47177 (19)	0.0811 (11)	
H14A	0.3321	0.3974	−0.5259	0.097*	
H14B	0.3616	0.1653	−0.4716	0.097*	
C15	0.30020 (19)	0.3870 (9)	−0.41962 (19)	0.0827 (11)	
H15A	0.3037	0.2558	−0.3779	0.099*	
H15B	0.257	0.3791	−0.4488	0.099*	
C16	0.31332 (17)	0.6412 (7)	−0.38706 (19)	0.0699 (9)	
H16A	0.2933	0.6627	−0.3386	0.084*	
H16B	0.2964	0.7743	−0.4249	0.084*	
H1N	0.5902 (10)	0.065 (4)	0.0092 (12)	0.018 (5)*	
H2N	0.6002 (12)	−0.213 (4)	0.0066 (14)	0.044 (7)*	
H4O	0.4439 (15)	0.568 (6)	−0.2807 (17)	0.060 (9)*	
H3O	0.5371 (13)	0.581 (6)	−0.1818 (14)	0.062 (9)*	
H1O	0.627 (3)	−0.029 (6)	0.250 (4)	0.23 (3)*	

### Atomic displacement parameters (Å^2^)

**Table:**

	*U*^11^	*U*^22^	*U*^33^	*U*^12^	*U*^13^	*U*^23^
Cl1	0.02064 (15)	0.01766 (14)	0.02759 (17)	0.00010 (16)	0.00212 (11)	0.00040 (17)
Br1	0.02064 (15)	0.01766 (14)	0.02759 (17)	0.00010 (16)	0.00212 (11)	0.00040 (17)
O1	0.0468 (8)	0.0297 (7)	0.0232 (7)	0.0082 (6)	−0.0050 (6)	−0.0016 (5)
O2	0.0360 (7)	0.0161 (5)	0.0271 (6)	0.0043 (5)	−0.0004 (5)	−0.0021 (5)
O3	0.0404 (8)	0.0446 (9)	0.0399 (9)	−0.0104 (7)	−0.0017 (7)	0.0096 (7)
O4	0.0491 (10)	0.0571 (10)	0.0427 (10)	−0.0191 (9)	−0.0137 (8)	0.0288 (8)
N1	0.0150 (5)	0.0144 (5)	0.0218 (6)	−0.0013 (7)	0.0011 (4)	−0.0021 (8)
C1	0.0136 (6)	0.0233 (9)	0.0234 (7)	−0.0005 (6)	0.0017 (5)	0.0009 (7)
C2	0.0182 (6)	0.0148 (6)	0.0214 (6)	−0.0010 (8)	−0.0008 (5)	0.0001 (8)
C3	0.0189 (7)	0.0184 (7)	0.0241 (8)	−0.0038 (6)	0.0018 (6)	−0.0020 (6)
C4	0.0170 (6)	0.0190 (10)	0.0302 (8)	0.0027 (5)	−0.0004 (6)	−0.0008 (6)
C5	0.0180 (8)	0.0337 (10)	0.0461 (12)	0.0008 (7)	0.0057 (8)	0.0089 (9)
C6	0.0255 (9)	0.0337 (11)	0.0439 (12)	0.0065 (8)	−0.0057 (8)	0.0047 (9)
C7	0.0586 (13)	0.0411 (15)	0.0388 (11)	−0.0170 (11)	0.0114 (10)	−0.0112 (10)
C8	0.075 (2)	0.0423 (14)	0.079 (2)	0.0001 (14)	0.0223 (16)	0.0113 (14)
C9	0.146 (4)	0.217 (7)	0.055 (2)	0.058 (5)	0.040 (2)	0.043 (3)
C10	0.082 (5)	0.067 (5)	0.053 (4)	−0.013 (3)	0.039 (4)	−0.018 (3)
C10B	0.075 (6)	0.197 (15)	0.097 (7)	0.001 (10)	0.043 (5)	−0.011 (10)
C11	0.0410 (14)	0.076 (2)	0.086 (2)	−0.0124 (14)	0.0061 (14)	0.0102 (18)
C12	0.0605 (15)	0.0477 (14)	0.0316 (11)	−0.0060 (12)	−0.0078 (10)	0.0176 (10)
C13	0.085 (3)	0.205 (6)	0.056 (2)	0.059 (3)	0.0158 (18)	0.038 (3)
C14	0.132 (3)	0.0586 (19)	0.0527 (17)	−0.0053 (19)	0.011 (2)	−0.0057 (14)
C15	0.097 (2)	0.085 (3)	0.0592 (18)	−0.005 (2)	−0.0168 (17)	0.0057 (18)
C16	0.075 (2)	0.071 (2)	0.0564 (17)	0.0054 (17)	−0.0213 (15)	0.0075 (16)

### Geometric parameters (Å, °)

**Table:**

O1—C3	1.317 (2)	C8—C9	1.466 (5)
O1—H1O	0.95 (2)	C8—H8A	0.99
O2—C3	1.200 (2)	C8—H8B	0.99
O3—C7	1.434 (3)	C9—C10	1.421 (9)
O3—H3O	0.939 (17)	C9—C10B	1.472 (12)
O4—C12	1.419 (2)	C9—H9A	0.99
O4—H4O	0.86 (3)	C9—H9B	0.99
N1—C1	1.4919 (18)	C10—C11	1.512 (8)
N1—C2	1.4986 (18)	C10—H10A	0.99
N1—H1N	0.88 (2)	C10—H10B	0.99
N1—H2N	0.925 (17)	C10B—C11	1.458 (11)
C1—C1^i^	1.513 (3)	C10B—H10C	0.99
C1—H1A	0.99	C10B—H10D	0.99
C1—H1B	0.99	C11—H11A	0.99
C2—C3	1.516 (3)	C11—H11B	0.99
C2—C4	1.544 (2)	C12—C16	1.491 (4)
C2—H2	1	C12—C13	1.510 (5)
C4—C5	1.517 (2)	C12—H12	1
C4—C6	1.522 (3)	C13—C14	1.622 (6)
C4—H4	1	C13—H13A	0.99
C5—H5A	0.98	C13—H13B	0.99
C5—H5B	0.98	C14—C15	1.458 (5)
C5—H5C	0.98	C14—H14A	0.99
C6—H6A	0.98	C14—H14B	0.99
C6—H6B	0.98	C15—C16	1.454 (5)
C6—H6C	0.98	C15—H15A	0.99
C7—C8	1.502 (4)	C15—H15B	0.99
C7—C11	1.533 (4)	C16—H16A	0.99
C7—H7	1	C16—H16B	0.99
			
C3—O1—H1O	108 (4)	C8—C9—H9A	109.4
C7—O3—H3O	108.9 (19)	C10B—C9—H9A	133.1
C12—O4—H4O	115 (2)	C10—C9—H9B	109.4
C1—N1—C2	112.98 (11)	C8—C9—H9B	109.4
C1—N1—H1N	104.2 (14)	C10B—C9—H9B	88.7
C2—N1—H1N	114.9 (14)	H9A—C9—H9B	108
C1—N1—H2N	109.0 (16)	C9—C10—C11	106.7 (4)
C2—N1—H2N	107.1 (17)	C9—C10—H10A	110.4
H1N—N1—H2N	108.5 (18)	C11—C10—H10A	110.4
N1—C1—C1^i^	108.99 (15)	C9—C10—H10B	110.4
N1—C1—H1A	109.9	C11—C10—H10B	110.4
C1^i^—C1—H1A	109.9	H10A—C10—H10B	108.6
N1—C1—H1B	109.9	C11—C10B—C9	106.9 (7)
C1^i^—C1—H1B	109.9	C11—C10B—H10C	110.3
H1A—C1—H1B	108.3	C9—C10B—H10C	110.3
N1—C2—C3	107.77 (15)	C11—C10B—H10D	110.3
N1—C2—C4	109.49 (12)	C9—C10B—H10D	110.3
C3—C2—C4	113.84 (14)	H10C—C10B—H10D	108.6
N1—C2—H2	108.5	C10B—C11—C7	106.2 (5)
C3—C2—H2	108.5	C7—C11—C10	102.7 (4)
C4—C2—H2	108.5	C10B—C11—H11A	129.4
O2—C3—O1	124.15 (16)	C7—C11—H11A	111.2
O2—C3—C2	123.18 (15)	C10—C11—H11A	111.2
O1—C3—C2	112.66 (14)	C10B—C11—H11B	86.8
C5—C4—C6	110.92 (15)	C7—C11—H11B	111.2
C5—C4—C2	112.68 (14)	C10—C11—H11B	111.2
C6—C4—C2	111.35 (15)	H11A—C11—H11B	109.1
C5—C4—H4	107.2	O4—C12—C16	109.6 (2)
C6—C4—H4	107.2	O4—C12—C13	112.1 (3)
C2—C4—H4	107.2	C16—C12—C13	103.6 (3)
C4—C5—H5A	109.5	O4—C12—H12	110.4
C4—C5—H5B	109.5	C16—C12—H12	110.4
H5A—C5—H5B	109.5	C13—C12—H12	110.4
C4—C5—H5C	109.5	C12—C13—C14	103.3 (3)
H5A—C5—H5C	109.5	C12—C13—H13A	111.1
H5B—C5—H5C	109.5	C14—C13—H13A	111.1
C4—C6—H6A	109.5	C12—C13—H13B	111.1
C4—C6—H6B	109.5	C14—C13—H13B	111.1
H6A—C6—H6B	109.5	H13A—C13—H13B	109.1
C4—C6—H6C	109.5	C15—C14—C13	105.6 (3)
H6A—C6—H6C	109.5	C15—C14—H14A	110.6
H6B—C6—H6C	109.5	C13—C14—H14A	110.6
O3—C7—C8	107.9 (2)	C15—C14—H14B	110.6
O3—C7—C11	110.5 (2)	C13—C14—H14B	110.6
C8—C7—C11	105.2 (2)	H14A—C14—H14B	108.8
O3—C7—H7	111	C14—C15—C16	104.6 (4)
C8—C7—H7	111	C14—C15—H15A	110.8
C11—C7—H7	111	C16—C15—H15A	110.8
C9—C8—C7	104.9 (3)	C14—C15—H15B	110.8
C9—C8—H8A	110.8	C16—C15—H15B	110.8
C7—C8—H8A	110.8	H15A—C15—H15B	108.9
C9—C8—H8B	110.8	C15—C16—C12	106.7 (3)
C7—C8—H8B	110.8	C15—C16—H16A	110.4
H8A—C8—H8B	108.8	C12—C16—H16A	110.4
C10—C9—C8	111.3 (4)	C15—C16—H16B	110.4
C8—C9—C10B	105.3 (7)	C12—C16—H16B	110.4
C10—C9—H9A	109.4	H16A—C16—H16B	108.6

Symmetry codes: (i) −*x*+1, *y*, −*z*.

### Hydrogen-bond geometry (Å, °)

**Table:**

*D*—H···*A*	*D*—H	H···*A*	*D*···*A*	*D*—H···*A*
N1—H1N···Cl1	0.88 (2)	2.37 (2)	3.253 (2)	175 (2)
N1—H2N···Cl1^ii^	0.93 (2)	2.32 (2)	3.209 (2)	161 (2)
N1—H2N···Br1^ii^	0.93 (2)	2.32 (2)	3.209 (2)	161 (2)
N1—H1N···Br1	0.88 (2)	2.37 (2)	3.253 (2)	175 (2)
O1—H1O···O4^iii^	0.95 (4)	2.50 (4)	3.446 (3)	172 (5)
O4—H4O···O3	0.86 (3)	1.89 (3)	2.728 (2)	165 (3)
O3—H3O···Cl1	0.94 (3)	2.29 (3)	3.204 (2)	163 (2)
O3—H3O···Br1	0.94 (3)	2.29 (3)	3.204 (2)	163 (2)
C1—H1A···O2	0.99	2.47	3.027 (2)	115
C1—H1B···Cl1^iii^	0.99	2.79	3.5460 (18)	134
C2—H2···O2^ii^	1.00	2.29	3.127 (2)	141
C6—H6C···O1	0.98	2.50	3.082 (3)	118

Symmetry codes: (ii) *x*, *y*−1, *z*; (iii) −*x*+1, *y*−1, −*z*.

## References

[bb1] Farrugia, L. J. (1997). *J. Appl. Cryst.* **30**, 565.

[bb2] Flack, H. D. (1983). *Acta Cryst.* A**39**, 876–881.

[bb3] Kaluderović, G. N., Gómez-Ruiz, S., Schmidt, H. & Steinborn, D. (2007). *Acta Cryst.* E**63**, o3491.

[bb4] Kaluderović, G. N., Heinemann, F. W., Knežević, N. Ž., Trifunović, S. R. & Sabo, T. J. (2004). *J. Chem. Crystallogr.* **34**, 185–189.

[bb5] Mistryukov, V. E., Mikhailov, Yu. N., Sergeev, A. V., Zhuravlov, M. G., Schelokov, R. N., Chernov, A. P., Fodorov, V. A. & Brekhovskikh, M. N. (1987). *Dokl. Akad. Nauk SSSR*, **295**, 1390–1393.

[bb6] Oxford Diffraction (2009). *CrysAlis CCD* and *CrysAlis RED* Oxford Diffraction Ltd, Abingdon, England.

[bb7] Schoenberg, L. N., Cooke, D. W. & Liu, C. F. (1968). *Inorg. Chem.* **7**, 2386–2393.

[bb8] Sheldrick, G. M. (2008). *Acta Cryst.* A**64**, 112–122. 10.1107/S0108767307043930 18156677

[bb9] Shkol’nikova, L. M., Ilyukhin, A. B., Gasparyan, A. V., Zavodnik, V. E., Poznyak, A. L. & Makarevich, S. S. (1990). *Kristallografiya*, **35***, *1421–1424.

[bb10] Shkol’nikova, L. M., Sotman, S. S., Poznyak, A. L. & Stoplyanskaya, L. V. (1992). *Kristallografiya*, **37**, 692–695.

[bb11] Shkol’nikova, L. M., Suyarov, N. D., Gasparyan, A. V., Poznyak, A. L., Zavodnik, V. E. & Dyaltova, N. M. (1989). *Zh. Strukt. Khim.* **30**, 92–104.

